# H5-based DNA constructs derived from selected highly pathogenic H5N1 avian influenza virus induce high levels of humoral antibodies in Muscovy ducks against low pathogenic viruses

**DOI:** 10.1186/1743-422X-11-74

**Published:** 2014-04-24

**Authors:** Olivier Guionie, Éric Niqueux, Michel Amelot, Stéphanie Bougeard, Véronique Jestin

**Affiliations:** 1 Anses (French Agency for Food, Environmental and Occupational Health & Safety), Ploufragan/Plouzané laboratory, Avian and Rabbit Virology Immunology and Parasitology Unit, B.P. 53, Ploufragan 22440, France; 2 European University of Britanny (Université européenne de Bretagne), Rennes, France; 3 Anses (French Agency for Food, Environmental and Occupational Health & Safety), Ploufragan/Plouzané laboratory, Avian Experimentation and Breeding Service, B.P. 53, Ploufragan 22440, France; 4 Anses (French Agency for Food, Environmental and Occupational Health & Safety), Ploufragan/Plouzané laboratory, Swine Epidemiology and Welfare Research Unit, B.P.53, Ploufragan 22440, France

**Keywords:** Avian influenzavirus, H5, DNA immunization, Duck, Hemagglutination inhibition, Virus neutralization, Cross-reactions

## Abstract

**Background:**

H5 low pathogenic avian influenza virus (LPAIV) infection in domestic ducks is a major problem in duck producing countries. Their silent circulation is an ongoing source of potential highly pathogenic or zoonotic emerging strains. To prevent such events, vaccination of domestic ducks might be attempted but remains challenging. Currently licensed vector vaccines derived from H5N1 HPAIV possess clade 0, clade 2.2 or clade 2.3.4 HA sequences: selection of the best HA candidate inducing the largest cross protection is a key issue. For this purpose, DNA immunization of specific pathogen free Muscovy ducks was performed using different synthetic codon optimized (opt) or native HA genes from H5N2 LPAIV and several H5N1 HPAIV clade 2.1, 2.2.1 and 2.3.4. Humoral cross-immunity was assessed 3 weeks after boost by hemagglutination inhibition (HI) and virus neutralization (VN) against three French H5 LPAIV antigens.

**Findings:**

Vaccination with LP H5N2 HA induced the highest VN antibody titre against the homologous antigen; however, the corresponding HI titre was lower and comparable to HI titres obtained after immunization with opt HA derived from clades 2.3.4 or 2.1. Compared to the other HPAIV-derived constructs, vaccination with clade 2.3.4 opt HA consistently induced the highest antibody titres in HI and VN, when tested against all three H5 LPAIV antigens and H5N2 LPAIV, respectively: differences in titres against this last strain were statistically significant.

**Conclusion:**

The present study provides a standardized method to assess cross-immunity based on HA immunogenicity alone, and suggests that clade 2.3.4-derived recombinant vaccines might be the optimal candidates for further challenge testing to vaccinate domestic Muscovy ducks against H5 LPAIV.

## Dedication

The final revision and preparation for publication of this report is dedicated to the memory of our late colleague Olivier Guionie, who untimely died on the 9^th^ of July 2013.

### Findings

Avian influenza virus (AIV) infection is a worldwide major concern for both animal and human health. Infection of domestic poultry by H5 and H7 highly pathogenic (HP) AIV has caused high mortality outbreaks in susceptible species and heavy economic losses following depopulation in infected areas. By contrast, AIV only rarely caused severe clinical signs or mortality following infection in domestic duck species. However, some of the reported outbreaks actually took place after evolution of the virus from low pathogenic (LP) to HP phenotype by nucleotide insertion in the hemagglutinin gene, following introduction and circulation of the virus in terrestrial domestic species [1]. To control and prevent silent AIV circulation, compulsory active surveillance of domestic bird flocks focused on H5 and H7 AIV has been implemented, and results from these serological surveys in the European Union show that domestic ducks and geese have the highest apparent seroprevalence for H5 and H7 subtypes [2]. Direct transmission of AIV from birds to humans has also been observed and results generally in mild infections with LPAIV H7, H9 and H10 subtypes, or severe and frequently fatal disease with HPAIV H5N1 and recently LPAIV H7N9, which raises again concern for uncontrolled evolution of LPAIV towards potentially zoonotic emerging strains (or even panzootic if the virus can acquire through mutation or reassortment an ability to transmit easily between humans) [3,4]. To prevent such events in the context of high prevalence of subclinical infection in duck-producing countries, reduction of LPAIV transmission between highly receptive domestic ducks would be essential and may be achieved using vaccination, in addition to biosecurity measures [5]. For LPAIV infection control in ducks, inactivated whole virion vaccines would have drawbacks: the vaccinal immune response is delayed and does not allow any easy differentiation from a post-infectious immune response (in the face of various circulating strains). On the contrary, recombinant hemagglutinin-derived vector-based vaccines are live vaccines that may allow a more rapid onset of immunity [6]. Since the AIV-specific post-vaccination immune response is directed against only one of AIV proteins, the hemagglutinin (HA), a straightforward strategy based on serological detection of antibodies against conserved internal antigens of AIV is also available to differentiate infected birds [7,8]. Most commercially available licensed recombinant vaccines were derived from H5N1 HPAIV and constructed using the original clade 0 A/goose/Guandong/96, a clade 2.3.4 or a clade 2.2 H5 insert sequence [8-10]. Two other licensed recombinant H5 influenza vaccines also exist but rely on respectively older or more phylogenetically distant strains: HPAIV H5N8 or LPAIV H5N2 HA sequences [11,12]. All the licensed vector vaccines mentioned above, which used Newcastle disease virus (NDV), fowlpox virus or turkey herpesvirus as a backbone, were extensively tested in chicken against different clades of H5N1 HPAIV [13-16]. However, to our knowledge their efficacy is not documented in domestic ducks against LPAIV.

In order to investigate which available recombinant vector vaccines would be efficient to vaccinate ducks against H5 LPAIV, a pre-screening strategy was used in this study, focusing on humoral immunity induced by DNA vaccination using H5 sequences. Several sequences were selected to retain only recent strains isolated from the first spread of Asian H5N1 HPAIV in 2003 and later on, which were subsequently used in licensed vector vaccines: these were clade 2.1, clade 2.2.1 and clade 2.3.4 H5 HPAIV strains (Table 1). Some of the tested sequences were artificially codon optimized to match chicken codon bias: previous studies had shown a positive effect of HA gene codon optimization on the induced antibody titres [17,18] and these findings were confirmed in the present study when comparing levels of antibodies induced against native and optimized clade 2.2.1 HPAIV sequences, in serological tests using a homologous H5 antigen (data not shown).

**Table 1:** DNA constructs and administration schemes used in immunization studies

**Group (number of ducks)**	**Treatment**^**(a)**^**/origin/clade-genotype**^**(b) **^**of the HA gene for DNA immunization**	**Accession number**^**(c) **^**and reference**
H5N2 LP (15)	N (LP H5N2)/A/duck/France/05057b/2005	AJ972673 [20]
Opt 2.1 (30)	Opt mut (HP H5N1)/A/chicken/Indonesia/7/03/2.1	ABO30346
2.2.1 G1 (15)	N mut (HP H5N1)/A/common pochard/France/06167/06/2.2.1 G1	AM498628 [22]
2.2.1 G2 (15)	N mut (HP H5N1)/A/swan/France/06299/06/2.2.1 G2^(d)^	EF395820 [22]
Opt 2.2.1 (15)	Opt mut (HP H5N1)/A/turkey/Turkey/1/05/2.2.1	ABD73284
Opt 2.3.4 (15)	Opt mut (HP H5N1)/A/duck/Laos/3295/06/2.3.4	ACH68553
Negative control (15)	No HA gene^(e)^	-

Prime immunization was done at five weeks and boost at eight weeks of age with 100 μg DNA per duck in all groups. Bleeding was done three weeks after the boost.

The percentages of amino acid identity and the corresponding differences between all six H5 sequences are displayed in Additional file 1: Tables S2 and S3.

^(a)^N = native; opt = chicken codon optimized; mut = mutated cleavage site HP → LP.

^(b)^When relevant.

^(c)^Genbank.

^(d)^The most represented subgroup in 2006 French outbreaks.

^(e)^Empty pcDNA3.1(-).

One hundred and twenty 5-week-old SPF Muscovy ducks were split in seven groups immunized with different plasmid DNA preparations, as described in Table 1, following a homologous prime-boost scheme. DNA was administered in the external thigh muscle with a needle-free insulin injection Medi Jector Vision system (Medi-Ject Corporation, Minneapolis, USA) as already described [19]. Animal experiment procedures on duck vaccination and protection studies were submitted to the French public regulatory control body, and approved by the French Ministry of agriculture, in accordance with European directives and national regulations. The six viral H5 sequences detailed in Table 1 were cloned in the pcDNA 3.1(-) vector (Invitrogen) and were selected from: (i) one LP H5N2 French strain [20,21] showing large cross-reactivity by hemagglutination inhibition [HI] test against French H5 LPAIV, which serves as a reference in the screening study; (ii) two HP H5N1 clade 2.2.1 viruses isolated in France in 2006 [22], as native sequences; (iii) three prototype HP H5N1 strains belonging to three different clades (selected as explained above), whose sequence were codon optimized for chicken usage and synthesized by Geneart AG (Germany). The original cleavage sites of the native and codon optimized (opt) HPAIV H5 sequences were modified as a LP site. Sera from the immunized Muscovy ducks were collected three weeks after boost and were tested against French H5 LPAIV strains in HI test following international standards [23] and in virus neutralization (VN) test using an adapted technique [24]. For HI tests, a technical detection limit of 2 log_2_ titre was achieved, and the OIE-recommended positivity threshold of 4 log2 was used to interpret results [23]. For VN tests, the detection limit was at 3 log_2_ titre, and no consensus positivity threshold exists. H5N2 A/duck/France/05057b/2005 was used in both VN and HI tests, whereas H5N1 A/duck/France/05066b/2005 and H5N3 A/duck/France/070090b/2007 were used in HI test only: these LP viruses were isolated from French domestic duck flocks and selected as representative of circulating French H5 LPAIV strains according to cross reactivity patterns against reference sera [20,21]. Percentages of amino acid identity and corresponding differences between H5 sequences used in the present study are displayed in Additional file 1: Tables S2 and S3.

Empty pcDNA 3.1(-) did not induce measurable HI and VN titres (data not shown), confirming all other measured titres were a direct result of the immune response elicited following expression in the ducks of the H5 inserted DNA constructs.

**Figure 1:**
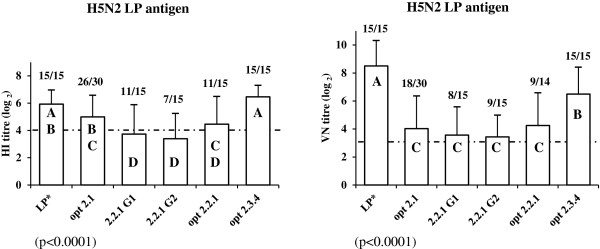
**Compared serological responses following immunization with different H5 DNA constructs.** HI test (left) and VN test (right) were performed against LP H5N2 A/duck/France/05057b/2005 [20,21]: geometric mean titres (log_2_) are displayed with corresponding standard deviations (error bar). A dashed line indicates the positivity threshold (≥4 log_2_) in HI test, and the detection threshold (≥3 log_2_) in VN test. The number of positive sera in HI test and of detected sera in VN test, over the total number of testable sera, is given above the corresponding bar for each group. For a given histogram, bars with different capital letters are statistically different, using Student-Newman-Keuls test following GLM procedure (SAS version 9.1, SAS Institute Inc., Cary, NC, USA) at risk α = 0.05. For the overall analysis of variance, p-values are mentioned in brackets above each histogram. For statistical analysis, sera with undetected antibody levels were given an arbitrary integer value immediately below the detection threshold of the respective test: respectively equal to 1 log_2_ and 2 log_2_ for HI and VN tests. *: HA in the DNA construct and in the antigen strain are homologous.

Vaccination with LP H5N2 HA induced positive HI antibody titres and detectable neutralizing antibodies in all vaccinated ducks, using the homologous LP antigen, with the highest mean VN titre (8.5 log_2_) compared to all tested HPAIV-derived HA (Figure 1). However, the corresponding mean HI titre (5.9 log_2_) using the same LP antigen was lower than the VN titre. The former was of intermediate value between HI titres obtained after vaccination with opt 2.3.4 HA and opt 2.1 HA, and was still significantly higher than HI titres obtained with native or optimized clade 2.2.1 HA (Figure 1).

Comparisons of serological response induced by vaccination schemes with the different selected HPAIV-derived DNA constructs showed that opt 2.3.4 HA immunization gave the highest serum antibody titres in both VN and HI tests against LP H5N2 antigen (6.5 log_2_ for both tests; Figure 1). All ducks vaccinated with opt 2.3.4 HA were also positive in HI test and had detectable neutralizing antibodies (Figure 1). For the last four constructs (opt 2.1 HA, opt 2.2.1 HA, 2.2.1 G1 HA and 2.2.1 G2 HA), VN and HI titres against LP H5N2 antigen, ranging respectively from 3.4 to 4.3 log_2_ and 3.4 to 5.0 log_2_, were significantly lower than titres induced by opt 2.3.4 HA. Accordingly to these differences in antibody titres, vaccination with these last four constructs did not constantly induce positive titres in HI test or detectable neutralizing antibodies: from nearly 10% ([4% - 31%] 95% confidence interval [CI]) to slightly more than 50% ([27% - 79%] 95% CI) of the vaccinated ducks were negative in HI or VN test against LP H5N2 antigen (Figure 1). However, HI titres obtained for opt 2.1 HA (5.0 log_2_) were not significantly different from homologous HI titres (induced by LP H5N2 HA).

**Figure 2:**
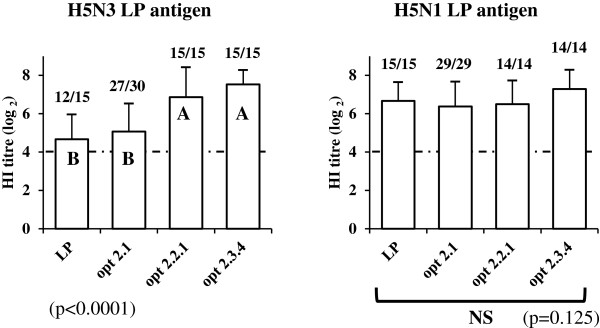
**Compared serological responses in HI tests, following immunization with different H5 optimized DNA constructs.** HI tests were performed against LP H5N1 A/duck/France/05066b/2005 and LP H5N3 A/Muscovy duck/France/070090b/2007 [20,21]: see Figure 1 for legend. NS indicates overall non statistically significant differences between groups.

HI tests performed against LP H5N3 (Figure 2) showed significantly higher HI titres obtained for opt 2.3.4 and opt 2.2.1 HA than for opt 2.1 and LP H5N2 HA (5.1 and 4.7 log_2_ respectively) and these differences were reflected in the number of positive sera in HI test: only following opt 2.3.4 and opt 2.2.1 HA vaccination were all ducks positive. HI tests against LP H5N1 (Figure 2) did not show any overall significant differences in antibody titres between the three optimized HP HA and LP H5N2 HA and all vaccinated ducks were positive irrespective of the DNA construct used: HI titres ranged from 6.4 to 7.3 log_2_, but the highest observed mean titre was obtained following opt 2.3.4 HA immunization.

In this study, the broadness of antibody response in SPF Muscovy ducks, following immunization with DNA encoding several H5 genes from different pathotypes and origins, was assessed so that the best insert could be determined on this criterion. This approach helps overcome interference of other AI proteins often encountered when extrapolating HA-induced cross immunogenicity results from sera prepared following immunization with whole virions. DNA immunization would better mimic vaccination with a viral vector expressing HA alone. This limitation to humoral immunity testing alone was implemented since the present DNA vaccination study would only serve to screen different H5 inserts to limit any further full challenge protection studies only to the relevant H5 sequence inducing the broadest cross-reactive antibodies.

Ducks generally develop poor HI systemic antibody response to experimental and natural AI infection, notably with H5 LPAI [25-27] (and unpublished data from the French NRL for avian influenza), with few individual antibody responses and low titres close to the positive threshold. Antibody titres against influenza were also highly variable in a post-vaccination surveillance study in France in 2006. Following vaccination with an inactivated H5N2 vaccine in free-range domestic ducks, sera were collected in flocks where unvaccinated sentinel birds had remained negative and were tested against the vaccinal antigen: HI titres ranged between 0 and 9 log_2_ with a mean titre of 6 log_2 _[28]. Other experimental studies have shown that vaccination against influenza in ducks with inactivated vaccines eventually requires higher antigen doses and multiple injections to be immunogenic and protective [29]. Depending on the type of vaccine used (inactivated or recombinant vector vaccine), experimental post-vaccination mean HI antibody titres against an antigen homologous to the vaccine usually remain low in Muscovy ducks, ranging from < 2 to 5.4 log_2_ following a single injection [30-32]. These titres could in some instances be raised following boost injection to moderate or high values of 3.4 to 7.5 log_2 _[30,31]. This could be due to a distinctive feature of duck immunoglobulin (Ig) Y (bird IgG equivalent) that co-exists as full-length IgY and Fc-truncated IgY(ΔFc), with variable ratios according to the individual and time of the immune response [33]. HI titre variability between ducks might correspond to the variable switch from full-length IgY to the truncated form: lack of Fc could potentially decrease steric hindrance of IgY that may be necessary for the inhibition of hemagglutination.

However, quality of the humoral immune response could also be influenced by the way vaccine antigen uptake by dendritic cells takes place and by the more or less efficient presentation to immune effector cells. DNA vaccines may actually stimulate the immune system in a more uniform and balanced way, and elicit less variable antibody titres. The level itself of obtained HI titres is comparable to previous experimental studies with classical inactivated or vector vaccines, since the present prime-boost DNA immunization model with a specific needle-free injection device was able to induce HI antibodies in most ducks with a mean titre around 6 log_2_ following LP H5N2 HA immunization. Indeed, all ducks vaccinated with this HA were positive in HI test against the homologous LP H5N2 antigen or a heterologous LP H5N1antigen, and a majority of them (12 out of 15) were positive in HI test against a LP H5N3 antigen (Figures 1 and 2). Homogeneous levels of antibody titres were also obtained, ensuring a low standard deviation between ducks of the same group and allowing comparison between immunogenic DNA inserts. However, boost injection was essential, as titres obtained with only a prime were too low and did not allow comparisons to be made between DNA constructs. Determination of an optimal dose of injected DNA was also influent on the level of antibody titres: prime-boost injections of different doses of LP H5N2 HA DNA construct (50, 100, 150, 200 and 250 μg) were tested. Mean HI titres against LP H5N2 antigen were significantly smaller for the 50 μg dose compared to the other groups, which had similar titres (data not shown): a 100 μg dose was therefore kept as optimal for the present study. Finally, a good correlation was also observed between HI and VN tests against LP H5N2 antigen (Pearson correlation coefficient = 0.72) with VN giving similar or significant higher titres (1.1 log_2_) than HI, strengthening the extrapolation of cross-immunogenicity results based on HI tests and their interpretation as possible cross-protection. Such data demonstrate the interest of DNA immunization for exploring both aspects and assessing cross-immunity based on HA immunogenicity alone.

A positive effect of codon optimized HA gene on antibody titres in immunized birds was found in previous studies [17,18]. However, in the present study, no statistically significant effect of codon optimization on cross-immunogenicity against a distant LP H5N2 antigen was observed for clade 2.2.1 HA: there was only a slight increase of at least 0.8 log_2_ and 0.7 log_2_ in respectively HI and VN test between native and codon optimized HA gene immunization.

Given the available licensed vector H5 vaccines, DNA inserts derived from clade 2.3.4 HPAIV appear to be an optimal choice if reduction of LPAIV infection is sought as an additional goal in vaccinating domestic ducks. Based on serological tests against three different representative French H5 LPAIV strains, DNA immunization with opt 2.3.4 consistently induced either the highest HI antibody titres (against a LP H5N2 antigen) or at least equivalent HI titres (against LP H5N1 and LP H5N3 antigens) compared to other tested HPAIV-derived HA. However, the choice of a relevant H5 insert is not the only way to optimize vaccination against LPAIV in ducks. Since LPAIV have a marked intestinal tropism in aquatic birds, it would also probably be valuable to develop and test vector vaccines based on enterotropic viruses. Notably, a recently devised duck enteritis virus (DEV) bearing a clade 2.3.4 H5 insert would be a relevant candidate [34]. DEV has a strong intestinal tropism and induces latent reactivating infections: both features would probably further enhance local immune responses in ducks. However, this vaccine is still experimental and is not available for direct infectious challenge tests to validate our present screening study.

## Competing interests

The authors declare that they have no conflict of interests.

## Author’s contributions

OG and VJ devised the original idea and design of this study. OG, VJ and MA organized and performed the experimental animal studies. OG and EN conducted laboratory assays and data interpretation. SB performed the statistical analysis of data. OG and VJ discussed and wrote initial versions of the report. EN and VJ were responsible for extensive final reworking of the manuscript. EN, MA, SB and VJ read and approved the final manuscript.

## Supplementary Material

Additional file 1: Table S2 Percentage of amino acid identity between different H5 sequences used in immunization studies and in serological test antigens. **Table S3.** Amino acid differences between H5 sequences used in immunization studies and in serological test antigens, according to their position. Click here for file
