# Supplementary material for: H5-based DNA constructs derived from selected highly pathogenic H5N1 avian influenza virus induce high levels of humoral antibodies in Muscovy ducks against low pathogenic viruses
Source: Virol J. 2014 Apr 24;11:74. doi: 10.1186/1743-422X-11-74 (PMC4029828; doi:10.1186/1743-422X-11-74)
Supplement: Additional file 1: Table S2 — Percentage of amino acid identity between different H5 sequences used in immunization studies and in serological test antigens. Table S3. Amino acid differences between H5 sequences used in immunization studies and in serological test antigens, according to their position. [file 1743-422X-11-74-S1.pdf]

Supplementary Table 2: Percentage of amino acid identity between different H5 sequences used in immunization studies and in serological test antigens.

	H5N2 LP	H5N1 LP	H5N3 LP	Opt 2.1	2.2.1 G1	2.2.1 G2	Opt 2.2.1
**H5N1 LP**	96,6						
**H5N3 LP**	97,5	96,6					
**Opt 2.1**	93,6	92,6	92,7				
**2.2.1 G1**	92,4	91,8	92,7	96,6			
**2.2.1 G2**	92,7	92,2	93,1	97,0	99,3		
**Opt 2.2.1**	92,9	92,4	93,1	97,0	98,9	99,3	
**Opt 2.3.4**	92,7	91,7	92,4	96,8	96,3	96,6	96,4

Supplementary Table 3: Amino acid differences between H5 sequences used in immunization studies and in serological test antigens, according to their position.

Only positions presenting difference(s) between H5N2 LP A/duck/France/05057b/2007 hemagglutinin and the other proteins are presented.

(.): for a given position (head of the column), amino acid identical to H5N2 LP protein.

	5	9	10	11	16	51	52	59	61	87	99	100	102	104	110	111	123	124	135	140
**H5N2 LP**	**V**	**A**	**I**	**V**	**S**	**K**	**A**	**S**	**N**	**L**	**D**	**N**	**V**	**G**	**D**	**L**	**S**	**T**	**R**	**N**
H5N1 LP	.	V	T	.	G	R	T	.	.	.	.	S	.	.	.	F	.	.	.	.
H5N3 LP	.	.	.	I	.	.	.	.	.	.	.	.	.	.	.	F	.	.	.	.
Opt 2.1	.	.	.	.	.	.	T	D	D	I	A	.	A	D	N	.	R	I	K	D
2.2.1 G1	.	.	.	.	.	.	T	D	D	.	I	.	A	D	N	F	R	I	K	D
2.2.1 G2	M	.	.	.	.	.	T	D	D	.	I	.	A	D	N	F	R	I	K	D
Opt 2.2.1	.	.	.	.	.	.	T	D	D	.	I	.	A	D	N	F	R	I	K	D
Opt 2.3.4	.	.	.	.	.	.	T	D	D	I	A	.	A	D	N	F	R	I	K	D

	142	145	152	154	156	157	170	171	172	179	190	194	197	204	205	211	214	223	225	228
**H5N2 LP**	**D**	**S**	**L**	**N**	**K**	**S**	**N**	**N**	**A**	**S**	**V**	**I**	**P**	**T**	**K**	**T**	**V**	**H**	**S**	**E**
H5N1 LP	.	.	P	.	R	.	.	S	.	.	.	.	.	.	.	N	.	Q	.	.
H5N3 LP	.	.	P	.	R	.	.	.	.	N	.	.	.	.	.	.	.	Q	.	.
Opt 2.1	E	.	P	Q	.	.	.	S	.	.	.	.	.	.	R	.	I	Q	L	K
2.2.1 G1	E	.	P	Q	R	.	D	.	.	.	.	V	.	I	R	.	I	Q	L	K
2.2.1 G2	E	.	P	Q	R	.	D	.	.	.	.	.	.	.	R	.	I	Q	L	K
Opt 2.2.1	E	A	P	Q	R	.	D	.	.	.	.	.	.	.	R	.	I	Q	L	K
Opt 2.3.4	E	.	P	Q	T	P	.	.	T	.	I	.	S	.	.	.	I	Q	L	K

	231	233	234	243	250	256	268	273	277	279	281	284	285	298	325	326	336	338	339	341
**H5N2 LP**	**T**	**P**	**K**	**E**	**R**	**N**	**Y**	**V**	**D**	**A**	**I**	**G**	**L**	**M**	**D**	**R**	**V**	**Q**	**R**	**T**
H5N1 LP	.	.	.	.	K	S	.	I	.	.	M	.	.	.	.	.	.	.	.	.
H5N3 LP	.	.	R	.	K	.	.	.	N	.	M	.	.	.	.	.	.	.	K	A
Opt 2.1	I	S	.	.	K	.	.	.	.	.	M	E	.	.	N	.	S	.	.	.
2.2.1 G1	.	S	.	.	K	.	N	.	.	T	M	E	.	I	N	.	S	.	G	K
2.2.1 G2	.	S	.	.	K	.	N	.	.	T	M	E	.	I	N	.	S	.	G	K
Opt 2.2.1	.	S	.	.	K	.	N	.	.	T	M	E	.	I	S	.	S	.	G	.
Opt 2.3.4	.	S	.	D	K	.	.	.	.	.	.	E	V	I	N	K	S	L	.	-

	374	390	393	410	485	494	500	509	511	523	544	545	550
**H5N2 LP**	**S**	**I**	**K**	**K**	**K**	**V**	**D**	**R**	**N**	**M**	**M**	**I**	**F**
H5N1 LP	.	.	R	.	.	.	.	K	.	.	.	V	.
H5N3 LP	T	.	.	.	.	.	.	.	.	.	.	V	.
Opt 2.1	.	V	.	R	.	I	N	.	K	I	.	M	L
2.2.1 G1	.	V	.	R	R	.	.	.	K	I	I	V	L
2.2.1 G2	.	V	.	R	R	.	.	.	K	I	.	V	L
Opt 2.2.1	.	V	.	R	R	.	.	.	K	I	.	V	L
Opt 2.3.4	.	V	.	R	.	.	.	.	K	I	.	V	L
